# Association between statin use and open-angle glaucoma: a nested case–control study using the Japanese claims database

**DOI:** 10.1038/s41598-023-38957-2

**Published:** 2023-07-19

**Authors:** Satoshi Yokoyama, Chihiro Nakagawa, Kouichi Hosomi

**Affiliations:** Division of Drug Informatics, School of Pharmacy, Kindai University, 3-4-1 Kowakae, Higashiōsaka City, Osaka 577-8502 Japan

**Keywords:** Epidemiology, Eye diseases

## Abstract

The association between statins and open-angle glaucoma (OAG) remains controversial. This study investigated the relationship between statins and OAG in Japanese patients with dyslipidemia using the Japanese administrative claims database. A nested case–control study using two models was conducted using the JMDC claims database (01/2005–01/2020). The onset of OAG: index date was defined as the diagnosis of glaucoma, prescription of anti-glaucoma drugs, or surgery of glaucoma. For each case, a maximum of 10 age-, sex-, and calendar year/month–matched controls were randomly selected by risk-set sampling with replacement. The number of statin prescriptions during the exposure assessment period, which was identified as the 12-month (model 1) or 24-month (model 2) periods prior to the index date, was used as an indicator for statin exposure. Adjusted odds ratios (aORs) and 95% confidence interval (CI) were estimated using conditional logistic regression analyses. We identified 375,373 patients with newly diagnosed dyslipidemia. Of these, 6180 cases and 61,792 controls (model 1) and 4153 cases and 41,522 controls (model 2) were selected. Statin use was not identified as a significant risk factor for OAG (model 1: aOR 0.98, 95% CI 0.93–1.03, model 2: aOR 0.97, 95% CI 0.91–1.04). Compared with nonexposure, short-term exposure (< 2 years) to statins was not related to an increased risk of OAG in the Japanese working-age population with dyslipidemia.

## Introduction

Glaucoma is a group of eye conditions that narrow the visual field due to optic neuropathy, and can cause visual impairment and irreversible blindness. The prevalence of glaucoma among Caucasians and Asian Americans is 2.8% and 3.5%, respectively^1^. When it comes to the Japanese population, the Tajimi study showed that the estimated prevalence of glaucoma in Japanese people older than 40 years was 3.9%^2^, a percentage that should certainly not be overlooked. Once diagnosed with an optic nerve or visual field disorder, the patients’ quality of life is significantly impacted. Therefore, early detection of glaucoma is very essential in suppressing disease progression and optimizing the subsequent therapeutic treatment. However, early detection of this disease requires a better understanding of the risk factors that are associated with the development of glaucoma.

Primary glaucoma can be divided into open-angle glaucoma (OAG), which is the most common form of glaucoma, and angle-closure glaucoma. Older age, elevated intraocular pressure, and high myopia have been known as risk factors in the pathogenesis of primary OAG^3^; and these risk factors can be applicable to the Japanese people as well^4^. A recent study showed the association between statin use and increased risk of OAG^5^, whereas other studies reported a decreased risk^6,7^. Some studies have reported that statin use was not associated with the risk of OAG^8,9^. Several statins are in clinical use, and the lipid profiles and lipid-lowering potency differ among statins. Yuan et al.^10^ reported that rosuvastatin users were more likely to suffer from glaucoma compared with users of other statins. However, Ooba et al.^11^ reported that the risk of OAG did not vary by type of statin in the Japanese working-age population. Therefore, the true relationship between statins and OAG remains unknown.

In this study, we investigated the relationship between statins and OAG in Japanese working-age patients with dyslipidemia using a database of administrative claims from the Japan Medical Data Center (JMDC).

## Method

### Data source

The JMDC administrative claims database is a large and chronologically organized database (JMDC Inc., Tokyo, Japan) that uses standardized disease classification and anonymous record linkage^12^. This database includes information from employment-based health insurance claims regarding employees and their dependents who are less than 75 years old. In total, the data of approximately 14 million insured persons in Japan were registered in this database between January 2005 and November 2021. The registered data for each patient included the following: age, sex, dates of hospital or clinic visits, International Statistical Classification of Diseases and Related Health Problems-Tenth Revision (ICD-10) codes, and prescription of drugs categorized according to the Anatomical Therapeutic Chemical (ATC) classification of both the European Pharmaceutical Market Research Association (EphMRA) and the World Health Organization. An encrypted personal identifier was used to link the claims data from various hospitals, clinics, and pharmacies.

### Study design

**Figure 1:**
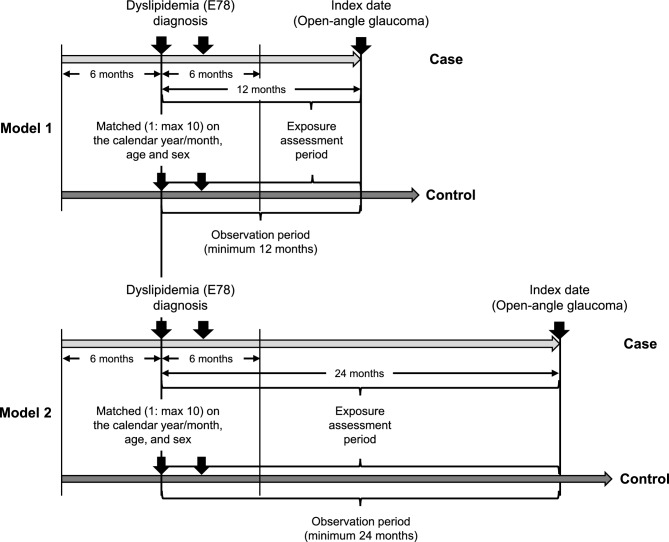
Study design and matching. In model 1, the exposure assessment period was set to 12 months before the index date. In Model 2, the exposure assessment period was set to 24 months prior to the index date. The first quartile (Q1) or the second quartile (Q2) of the number of statin prescriptions in the cohort during the exposure assessment period was calculated and the exposure was defined as follows; Q1 exposure: the number of statin prescription ≥ Q1, Q2 exposure: the number of statin prescription ≥ Q2.

We conducted a nested case–control study using the JMDC claims database (January 2005–January 2020). When investigating the effects of time-dependent exposures on rare outcomes in very large databases, a nested case–control design is a useful alternative for cohort design^13^. Figure 1 shows the study design and matching. Our study used two model cohorts to evaluate the robustness of the results. First, we identified newly diagnosed patients with dyslipidemia (ICD-10 code: E78) who had already been included in the JMDC database for at least six months before the first diagnosis. Furthermore, we selected patients who had been diagnosed once or more within six months after the first diagnosis of dyslipidemia in order to select patients who had continuously received medical examination and treatment. We excluded patients under 20 years of age, patients whose onset of OAG (outcome) was before the first diagnosis of dyslipidemia, and patients who did not have a minimum of 12 months (model 1) or 24 months (model 2) of participation after the first diagnosis of dyslipidemia. Because glaucoma represents a latent progression, it is necessary to consider the duration of exposure when investigating the effect of statins on glaucoma. In this study, we created two models to investigate the effect of statin exposure duration. Observation periods were defined as periods from the first diagnosis of dyslipidemia to onset of outcome (index date) or the final diagnosis of dyslipidemia, whichever came first. Within these cohorts, we identified all cases with outcomes during the observation periods. Consequently, we constructed a risk set of possible controls for each case with at least an equal duration of observation periods at the case’s index date. Cases with outcomes were randomly matched to a maximum of 10 controls according to age (± 5 years), sex, and calendar year/month (± 3 months) of the date of first dyslipidemia diagnosis through risk-set sampling. Controls were selected with replacement, which implies that controls are randomly selected longitudinally throughout the risk period and are chosen to represent risk time. The same index date of the matched case was applied to comparator controls. This study was approved by the Ethics Committees of the Kindai University School of Pharmacy on April 14, 2018 (approval number, 18-128). The requirement for informed consent was waived due to the anonymous nature of the data.

### Outcome

The onset of OAG in patients with dyslipidemia was defined as the outcome of this study, and the index date of case was defined as the onset of OAG. To define the onset of OAG, we used a composite outcome measure. In models 1 and 2, OAG was identified based on the diagnosis code of glaucoma (ICD-10: H401, H406, and H409), prescribed anti-glaucoma drugs (EphMRA ATC code: S1E2), or a Japanese medical procedure code of glaucoma surgery (K268).

### Exposure

Six HMG-CoA reductase inhibitors, including rosuvastatin, atorvastatin, pitavastatin, pravastatin, simvastatin, and fluvastatin were defined as statins in our study. We investigated exposure to statins during a 12- or 24-month period prior to the index date in models 1 and 2, respectively (Fig. 1). The number of statin prescriptions during the exposure assessment period was totaled, and the number of the first (Q1) and second (Q2) quartiles was calculated by descriptive statistics. Patients who were prescribed for Q1 or more times (Q1 exposure) or Q2 or more times (Q2 exposure) were considered to have exposure to statins. A total of four patterns were analyzed for Q1 and Q2 exposures in the models 1 and 2.

### Covariates

We examined age, sex, and comorbidities at the first diagnosis of dyslipidemia. Diseases with an ICD-10 code of two or more times within six months prior to the first diagnosis of dyslipidemia were defined as comorbidities. Identified comorbidities that could influence the outcomes of this study included diabetes mellitus (defined as ICD-10 code E11 and E14), hypertension (I10), hypotension (I95), hyperuricemia/gout (E790 and M10), arrhythmias/heart failure (I48, I49, and I50), ischemic heart disease (I20-I25), cerebrovascular disease (I60-I69), transient ischemic attack (G45), migraines (G439), obstructive sleep apnea (G473), and myopia (H521). Infectious or inflammatory conditions, including Behçet’s disease (M352), congenital rubella (P350), cytomegalovirus retinitis (B258 and B259), herpetic disease (B00, B01, and B02), human leukocyte antigen B27-related disease (M02, M45, and L405), juvenile idiopathic arthritis (M080), Lyme disease (A692), rheumatoid arthritis (M05 and M06), sarcoidosis (D86), syphilis (A50-A53), systemic lupus erythematosus (M32), toxocariasis (B830) or toxoplasmosis (B58), systemic beta-blocker use (defined as EphMRA ATC code C7), and steroid use (defined as EphMRA ATC code H2, S1B, S3B, and S3C), were also identified as potential confounders with reference to previous studies^1,14^.

### Data analysis

Categorical variables were reported as number and percentage. Continuous variables were reported as median and interquartile range [IQR]. Conditional logistic regression analyses were performed to estimate the odds ratio and its respective 95% confidence interval (CI). Conditional logistic regression method for nested case–control study with time-dependent covariates has superior computational efficiency because only a sample of all possible controls are included in the risk set of each case^13^. Crude odds ratios were adjusted by all variables: diabetes mellitus, hypertension, hypotension, hyperuricemia/gout, arrhythmias/heart failure, ischemic heart disease, cerebrovascular disease, transient ischemic attack, migraines, obstructive sleep apnea, myopia, infectious or inflammatory conditions, systemic beta-blocker use, and steroid use. Data management was performed using the Visual Mining Studio software (version 9.0; NTT DATA Mathematical Systems Inc., Tokyo, Japan) and SAS studio (version 3.8, Enterprise Edition; SAS Institute, Cary, NC). All statistical tests were two-sided, and a value of *p* < 0.05 was considered statistically significant using IBM SPSS 28 Statistics (IBM Inc., Armonk, NY).

## Results

### Study participants

**Figure 2:**
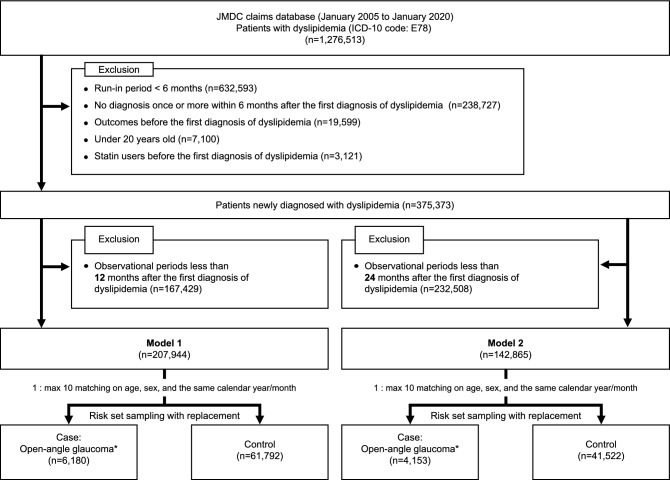
Flow diagram of patient selection. *Open-angle glaucoma based on the diagnosis code of glaucoma (ICD-10: H401, H406, and H409), prescribed anti-glaucoma drugs (EphMRA ATC code: S1E2), or a Japanese medical procedure code of glaucoma surgery (K268). ICD-10, International Statistical Classification of Diseases and Related Health Problems-Tenth Revision; EphMRA, the European Pharmaceutical Market Research Association; ATC, anatomical therapeutic chemical.

Figure 2 illustrates the flow diagram of patient selection. We identified 1,276,513 patients who had a diagnosis code of dyslipidemia in the JMDC claims database. Patients were excluded if they had < 6 months run-in period (n = 632,593), no new diagnosis of dyslipidemia once or more within six months after the first diagnosis (n = 238,727), outcomes before the first diagnosis of dyslipidemia (n = 19,599), were younger than 20 years old (n = 7,100), statin users before the first diagnosis of dyslipidemia (n = 3121), and had an observation period of less than 12 months after the first diagnosis of dyslipidemia (n = 167,429). After applying these criteria, 6180 cases and 61,792 controls were identified in model 1. Similarly, 4153 cases and 41,522 controls were identified in model 2.

Tables 1 and 2 shows the demographic and clinical characteristics of the cases and controls included in models 1 and 2, respectively. Male patients predominated (approximately 60%), and the median age was 51 years. The median [IQR] observational period after the first diagnosis of dyslipidemia in models 1 or 2 were 32 [20–53] or 45 [32–64] months, respectively. Approximately half of the patients diagnosed with dyslipidemia were prescribed statins at least once. In model 1, the Q1 and Q2 of the number of statin prescriptions were 5 and 7, suggesting more than 75% and 50% patients who received statins within a 12-month period prior to the index date and received Q1 exposure and Q2 exposure, respectively. In model 2, the Q1 and Q2 of the number of statin prescription was 8 and 13, suggesting more than 75% and 50% patients who received statins within 24 months prior to the index date and received Q1 exposure and Q2 exposure, respectively.

**Table 1:** Baseline characteristics in model 1.

Variables	Cases		Controls	
	n = 6180		n = 61,792	
Age (median [IQR])	52	[46–57]	51	[46–57]
Male, n (%)	3714	(60.1)	37,139	(60.1)
Observational periods after the first diagnosis of dyslipidemia (months, median [IQR])	32	[20–53]	32	[20–53]
Patients prescribed statins once or more during the exposure assessment period, n (%)	3029	(49.0)	30,979	(50.1)
The number of times of statin prescription within 12-month period prior to the index date (n, median [IQR])	8	[5–11]	7	[5–11]
Exposure				
Statin use (Q1 exposure), n (%)	2387	(38.6)	24,149	(39.1)
Statin use (Q2 exposure), n (%)	1755	(28.4)	17,083	(27.6)
Comorbidities				
Diabetes mellitus, n (%)	722	(11.7)	5595	(9.1)
Hypertension, n (%)	1474	(23.9)	13,508	(21.9)
Hypotension, n (%)	14	(0.2)	128	(0.2)
Hyperuricemia/gout, n (%)	362	(5.9)	3521	(5.7)
Arrhythmias/heart failure, n (%)	285	(4.6)	2260	(3.7)
Ischemic heart disease, n (%)	204	(3.3)	1613	(2.6)
Cerebrovascular disease, n (%)	162	(2.6)	1547	(2.5)
Transient ischemic attack, n (%)	19	(0.3)	117	(0.2)
Migraines, n (%)	96	(1.6)	787	(1.3)
Obstructive sleep apnea, n (%)	103	(1.7)	799	(1.3)
Myopia, n (%)	198	(3.2)	956	(1.5)
Infectious or inflammatory conditions, n (%)	162	(2.6)	1517	(2.5)
Comedications				
Systemic beta-blocker use, n (%)	209	(3.4)	2080	(3.4)
Steroid use, n (%)	768	(12.4)	5906	(9.6)

Statin use (Q1 exposure): five times or more prescriptions within 12-month period prior to the index date.

Statin use (Q2 exposure): seven times or more prescriptions within 12-month period prior to the index date.

IQR, interquartile range.

**Table 2:** Baseline characteristics in model 2.

Variables	Cases		Controls	
	n = 4153		n = 41,522	
Age (median [IQR])	51	[45–56]	51	[45–56]
Male, n (%)	2547	(61.3)	25,469	(61.3)
Observational periods after the first diagnosis of dyslipidemia (months, median [IQR])	45	[32–64]	45	[32–64]
Patients prescribed statins once or more during the exposure assessment period, n (%)	2245	(54.1)	22,785	(54.9)
The number of times of statin prescription within 24-month period prior to the index date (n, median [IQR])	13	[8–21]	13	[8–21]
Exposure				
Statin use (Q1 exposure), n (%)	1807	(43.5)	18,266	(44.0)
Statin use (Q2 exposure), n (%)	1240	(29.9)	12,217	(29.4)
Comorbidities				
Diabetes mellitus, n (%)	486	(11.7)	3769	(9.1)
Hypertension, n (%)	957	(23.0)	8834	(21.3)
Hypotension, n (%)	12	(0.3)	58	(0.1)
Hyperuricemia/gout, n (%)	228	(5.5)	2267	(5.5)
Arrhythmias/heart failure, n (%)	182	(4.4)	1468	(3.5)
Ischemic heart disease, n (%)	125	(3.0)	1084	(2.6)
Cerebrovascular disease, n (%)	99	(2.4)	965	(2.3)
Transient ischemic attack, n (%)	8	(0.2)	79	(0.2)
Migraines, n (%)	60	(1.4)	489	(1.2)
Obstructive sleep apnea, n (%)	62	(1.5)	491	(1.2)
Myopia, n (%)	129	(3.1)	640	(1.5)
Infectious or inflammatory conditions, n (%)	99	(2.4)	922	(2.2)
Comedications				
Systemic beta-blocker use, n (%)	143	(3.4)	1,335	(3.2)
Steroid use, n (%)	516	(12.4)	4,103	(9.9)

Statin use (Q1 exposure): eight times or more prescriptions within 24-month period prior to the index date.

Statin use (Q2 exposure): thirteen times or more prescriptions within 24-month period prior to the index date.

IQR, interquartile range.

### Association between statin use and OAG

Table 3 shows the odds ratios for OAG. Conditional logistic regression analysis indicated that statin use (Q1 exposure) was not identified as a significant risk factor in these two models (model 1: adjusted odds ratio [aOR] 0.98, 95% CI 0.93–1.03, model 2: aOR 0.97, 95% CI 0.91–1.04). Furthermore, Q2 exposure was evaluated in the same way, and statin use (Q2 exposure) was not identified as a significant risk factor in these two models (model 1: aOR: 1.03, 95% CI 0.97–1.09, model 2: aOR 1.01, 95% CI 0.94–1.09). Table 4 shows the association between Q1 or Q2 exposure of each statin and the onset of OAG. No statins were identified as risk factors for OAG onset.

**Table 3:** Odds ratios for open-angle glaucoma.

Statin use	Crude odds ratio	(95% CI)	Adjusted odds ratio*	(95% CI)	*p*-value
Model 1					
Statin use (Q1 exposure), n (%)	0.98	(0.93–1.04)	0.98	(0.93–1.03)	0.404
Statin use (Q2 exposure), n (%)	1.04	(0.98–1.10)	1.03	(0.97–1.09)	0.309
Model 2					
Statin use (Q1 exposure), n (%)	0.98	(0.92–1.05)	0.97	(0.91–1.04)	0.434
Statin use (Q2 exposure), n (%)	1.02	(0.95–1.10)	1.01	(0.94–1.09)	0.763

Q1 exposure in model 1: five times or more prescriptions within 12-month period prior to the index date.

Q2 exposure in model 1: seven times or more prescriptions within 12-month period prior to the index date.

Q1 exposure in model 2: eight times or more prescriptions within 24-month period prior to the index date.

Q2 exposure in model 2: thirteen times or more prescriptions within 24-month period prior to the index date.

*Adjusted for diabetes mellitus, hypertension, hypotension, hyperuricemia/gout, arrhythmias/heart failure, ischemic heart disease, cerebrovascular disease, transient ischemic attack, migraines, obstructive sleep apnea, myopia, infectious or inflammatory conditions, systemic beta-blocker use, and steroid use.

CI, confidence interval.

**Table 4:** Odds ratio of each statin for open-angle glaucoma.

Statin use	Model 1									Model 2								
	Cases		Controls		Crude odds ratio	(95% CI)	Adjusted odds ratio*	(95% CI)	*p*-value	Cases		Controls		Crude odds ratio	(95% CI)	Adjusted odds ratio*	(95% CI)	*p*-value
	n = 6180		n = 61,792							n = 4153		n = 41,522						
Q1 exposure																		
Rosuvastatin	920	(14.9)	9060	(14.7)	1.02	(0.95–1.10)	1.02	(0.94–1.10)	0.665	675	(16.3)	6807	(16.4)	0.99	(0.91–1.08)	0.98	(0.90–1.07)	0.666
Atorvastatin	638	(10.3)	6543	(10.6)	0.97	(0.89–1.06)	0.97	(0.89–1.06)	0.455	491	(11.8)	5127	(12.3)	0.95	(0.86–1.05)	0.94	(0.85–1.04)	0.252
Pitavastatin	494	(8.0)	4894	(7.9)	1.01	(0.92–1.11)	0.99	(0.90–1.10)	0.893	381	(9.2)	3600	(8.7)	1.06	(0.95–1.12)	1.04	(0.93–1.16)	0.540
Pravastatin	262	(4.2)	2686	(4.3)	0.97	(0.86–1.11)	0.98	(0.86–1.11)	0.708	200	(4.8)	2022	(4.9)	0.99	(0.85–1.15)	0.98	(0.84–1.14)	0.775
Simvastatin	35	(0.6)	465	(0.8)	0.75	(0.53–1.06)	0.75	(0.53–1.06)	0.101	26	(0.6)	365	(0.9)	0.71	(0.48–1.06)	0.71	(0.48–1.06)	0.095
Fluvastatin	43	(0.7)	331	(0.5)	1.30	(0.95–1.79)	1.26	(0.92–1.74)	0.157	29	(0.7)	313	(0.8)	0.93	(0.63–1.36)	0.90	(0.61–1.33)	0.600
Non-user	3814	(61.7)	37,972	(61.5)	Reference		Reference			2385	(57.4)	23,605	(56.8)	Reference		Reference		
Q2 exposure																		
Rosuvastatin	640	(10.4)	6252	(10.1)	1.03	(0.94–1.12)	1.03	(0.94–1.12)	0.504	441	(10.6)	4332	(10.4)	1.02	(0.92–1.13)	1.02	(0.92–1.13)	0.733
Atorvastatin	475	(7.7)	4551	(7.4)	1.05	(0.95–1.16)	1.04	(0.95–1.15)	0.393	339	(8.2)	3344	(8.1)	1.02	(0.90–1.14)	1.01	(0.90–1.14)	0.893
Pitavastatin	357	(5.8)	3405	(5.5)	1.05	(0.94–1.18)	1.04	(0.93–1.17)	0.497	244	(5.9)	2324	(5.6)	1.05	(0.92–1.21)	1.04	(0.91–1.20)	0.561
Pravastatin	191	(3.1)	1932	(3.1)	0.99	(0.85–1.15)	0.99	(0.86–1.17)	0.999	137	(3.3)	1320	(3.2)	1.04	(0.87–1.24)	1.04	(0.87–1.25)	0.645
Simvastatin	23	(0.4)	349	(0.6)	0.66	(0.43–1.01)	0.66	(0.43–1.01)	0.054	19	(0.5)	254	(0.6)	0.75	(0.47–1.19)	0.75	(0.47–1.20)	0.227
Fluvastatin	32	(0.5)	236	(0.4)	1.36	(0.94–1.97)	1.33	(0.91–1.93)	0.132	22	(0.5)	207	(0.5)	1.06	(0.68–1.66)	1.05	(0.68–1.64)	0.814
Non-user	4469	(72.3)	45,107	(73.0)	Reference		Reference			2956	(71.2)	29,782	(71.7)	Reference		Reference		

Q1 exposure: prescribed five times or more within 12-month period prior to the index date (model 1) or eight times or more within 24-month period prior to the index date (model 2).

Q2 exposure: prescribed seven times or more within 12-month period prior to the index date (model 1) or thirteen times or more within 24-month period prior to the index date (model 2).

*Adjusted for diabetes mellitus, hypertension, hypotension, hyperuricemia/gout, arrhythmias/heart failure, ischemic heart disease, cerebrovascular disease, transient ischemic attack, migraines, obstructive sleep apnea, myopia, infectious or inflammatory conditions, systemic beta-blocker use, and steroid use.

CI, confidence interval.

## Discussion

This nested case–control study used the Japanese administrative claims database to investigate the association between statin use and onset of OAG. Since studies using databases have several limitations, we constructed a total of four patterns in the two-model design to increase the robustness of our results. Consequently, our findings revealed that there was no significant association between short-term statin use (< 2 years) and onset of OAG in Japanese working-age population with dyslipidemia, a finding that was consistent across all models used in this study.

The National Health and Nutrition Survey in Japan (2018), reported by the Ministry of Health, Labour and Welfare, showed that the frequency of high low-density lipoprotein cholesterol levels decreased after peaking in the 40 s for men and in the 50 s for women^15^. The proportion of males in our study was approximately 60%, with a median age of 52 years, indicating that the JMDC database can reflect the portion of the Japanese population with dyslipidemia. Approximately half of the patients diagnosed with dyslipidemia were prescribed statins once or more in both cases and controls. The number of patients prescribed statins once or more times was higher in model 2 (≥ 24 months of observation) compared to model 1 (≥ 12 months of observation), suggesting that a long-term prevalence of dyslipidemia may lead to an increased number of statin users.

One of the advantages of our study is that it confirms the use of a nested case–control design for estimating the hazard ratio. In the nested case–control design, we randomly selected controls by risk-set sampling with replacement according to the index date of cases. The odds ratio obtained by analyzing the time point–matched data by conditional logistic regression correctly estimated the hazard ratio of the Cox regression^16^. Our results can be interpreted in that a 12- or 24-month period administration of statins to young patients with dyslipidemia does not make a significant difference in the hazard ratio of OAG compared to patients without administration of statins. A nested case–control study targeting elderly Australians also showed that statin use was not associated with glaucoma onset^10^. However, their study showed an increased risk of glaucoma onset in participants with a longer duration (> 3 years) of statin use compared with a shorter duration (< 1 year). No consistent conclusions have been extracted regarding the relationship between the duration of statin administration and the risk of OAG, although some reports have suggested that long-term or short-term statin use can reduce the respective risk^7,17^. Another large-scale study with a long follow-up duration of five or more years also showed that longer statin use was not associated with risk of OAG^8^. The twenty-four months period of exposure to statins was considered in our study; however, longer-term observations may need to be considered. Racial differences may influence the relationship between statin use and OAG. A cross-sectional study using big data in the United Kingdom showed that no evidence of a protective association between statin use and glaucoma was found^18^. In a study of the Korean population, dyslipidemia treatment was not found to be significantly associated with OAG^19^. Regardless of the racial or ethnic composition, statins are presumably not involved in the onset of OAG. No statin was found to be significantly associated with the onset of OAG in our results for the individual statin investigation. For simvastatin, the odds ratios in all the analyses were below 1.0, although not statistically significant. Simvastatin has been reported for its possible role in visual field stabilization in glaucoma patients^20^. Consideration with a larger sample size of simvastatin is desired.

Several reports have examined the relationship between serum cholesterol levels and glaucoma/intraocular pressure^21,22^. Serum HDL3 cholesterol level, which is involved in the promotion of cholesterol efflux and has a protective effect on vascular endothelium, was reported to be associated with glaucoma^9^. These reports indicate that dyslipidemia and blood lipid level are associated with glaucoma and high intraocular pressure. Regardless of the use or nonuse of statins in dyslipidemia, poor control of dyslipidemia may increase the risk of glaucoma depending on serum cholesterol level.

Comorbidities, such as diabetes, myopia, and the use of steroids, were identified as significant risk factors for OAG in our models (Supplementary Table S1), a finding which is consistent with current literature. Diabetes mellitus has already been suggested to increase the risk of OAG^23,24^, because higher serum glucose levels are associated with high intraocular pressure, which is thought to result in optic nerve damage^25^. Myopia, especially high myopia, has been reported as a major risk factor for OAG^26,27^. This is further supported by the Tajimi study conducted in Japan, which also showed an association between myopia and glaucoma^4^. Finally, steroid use has been long known to increase intraocular pressure, resulting in steroid-induced glaucoma^28–30^. Patients with these factors are at a particularly increased risk of developing OAG, thus regular examinations are extremely essential. Different results of the conditional logistic regression analysis were observed between models 1 and 2: hypertension and arrythmias/heart failure in model 1 and hypotension in model 2 were significantly associated with OAG. The reason for the different results might be the presence of unmeasured confounders. Thus, it is necessary to be careful when interpreting the results.

Nonetheless, our study has several limitations. First, since the JMDC administrative claims database is based on social insurance enrollees in the Japanese working-age population, most of the people registered is under 65 years of age, and thus data for patients over 75 years old are not included. This is especially important considering that the incidence of glaucoma increases with age^31^. Therefore, the results of this this study cannot be extrapolated to individuals older than 65 years. Furthermore, the exposure assessment period was set to a maximum of 24 months for the working-age population, which might have been too short. Further studies considering a longer exposure period and including elderly patients with dyslipidemia are needed. Second, OAG is associated with ocular features, such as intraocular pressure and family history. However, this information was not included in the JMDC claims database. Serum cholesterol level was also not included. Therefore, these factors could not be considered in our analysis. Furthermore, our definition might not accurately identify primary OAG. Glaucoma is likely heterogenous (OAG, exfoliative glaucoma, some secondary glaucoma, and some ocular hypertension). Since using K268 medical procedure code of glaucoma surgery to define the outcome, we could not rule out the possibility that a few patients with angle-closure glaucoma were included in the cases. There were six and three cases with K268 in models 1 and 2, respectively. Surgery is usually conducted for angle-closure glaucoma rather than OAG. Therefore, there would have been misclassification of the outcome in this study. Third, the number of prescriptions for statins was used as an indicator of exposure. However, factors such as adherence to statins, daily dose, or dose intensity were not taken into consideration. Furthermore, modifiable environmental factors, such as lifestyle, exercise, and nutrition^32^, could not be investigated. Fourth, patients diagnosed once or more within six months after the first dyslipidemia diagnosis were eligible for this study. Selection bias might have occurred when selecting participants.

## Conclusion

In conclusion, our analysis demonstrated that no significant association was observed between short-term statin usage (< 2 years) and the onset of OAG among the Japanese working-age population with dyslipidemia.

## Supplementary Information

Supplementary Table S1.

## Data Availability

The datasets generated and analyzed during the current study are available from the corresponding author on reasonable request.
